# Linking vector favourable environmental conditions with serological evidence of widespread bluetongue virus exposure in livestock in Ecuador

**DOI:** 10.1038/s41598-025-95918-7

**Published:** 2025-04-24

**Authors:** Alfredo Acosta, Maritza Barrera, David Jarrín, Alexander Maldonado, Johanna Salas, Guilherme Camargo, Beatriz Mello, Alexandra Burbano, Euclides DelaTorre, Bernd Hoffman, Klaas Dietze

**Affiliations:** 1 Friedrich-Loeffler-Institut, Greifswald, Germany; 2 Preventive Veterinary Medicine Department, School of Veterinary Medicine, University of São Paulo, São Paulo, Brazil; 3 Agencia de Regulación y Control Fito y Zoosanitario-Agrocalidad, Quito, Ecuador; 4 Veterinary Department, Faculty of Veterinary Sciences, Universidad Técnica de Manabí, Portoviejo, Ecuador; 5 Department of Epidemiology, Disease Surveillance and Risk Assessment, Swedish Veterinary Agency, SVA, Ulls väg 2B, 75189 Uppsala, Sweden

**Keywords:** Bluetongue, Surveillance system, Culicoides, Risk analysis, Policy, Ecological epidemiology, Ecological modelling, Tropical ecology, Agroecology

## Abstract

Despite existing knowledge of bluetongue disease (BT) in Latin America, little information is available on its actual spread and overall burden. As a vector-borne disease, high-risk areas for BT coincide with environmental conditions favourable for the prevailing vector. In Ecuador, information on the presence of BT is limited to singled out virological findings. In this study, we obtained serological evidence for BT virus exposure from the passive surveillance system of the National Veterinary Service, which monitors reproductive-vesicular diseases, including FMD and BT, as part of differential diagnosis. Bioclimatic factors relevant to *Culicoides* development as the main vector and host abundance at the parish level were considered as risk factors and analysed using a logistic regression model. The results reveal widespread evidence of bluetongue virus exposure, geographically aligning with favourable vector ecosystems within a temperature range of 12–32 °C. Key variables for predicting high-risk BT areas include cattle population, maximum temperature of the warmest month, minimum temperature of the coldest month, temperature seasonality, and precipitation of the driest month. This analysis, the first of its kind for an Andean country with diverse ecosystems, provides a foundation for initial strategic approaches for targeted surveillance and control measures, considering a One Health approach.

## Introduction

Bluetongue disease BT in Latin America remains relatively underexplored, with limited information on its spread. Given its vector-borne nature, BT prevalence is expected to align with favourable environmental conditions for its primary vector. In Ecuador, knowledge on BTV presence has been limited, highlighting a gap in comprehensive surveillance. We hereby aim to provide scientific grounds for future targeted surveillance and control strategies.

Bluetongue (BT) is a noncontagious disease affecting domestic and wild ruminants caused by the bluetongue virus (BTV), which belongs to the genus *Orbivirus* in the *Reoviridae* family. The virus is capable of infecting cattle, sheep, deer, goats and camelids^1,2^ and is transmitted by insects from the genus *Culicoides,* as the disease causes substantial economic losses and is a major concern for international trade^3^, it is notifiable to the World Organization for Animal Health (WOAH). Many countries have adopted regulatory control measures addressing bluetongue; however, the effectiveness of these measures depends on the capacities and resources of their veterinary services, including laboratory facilities^4^.

The disease has multiple manifestations that depend on the host and viral factors, and its clinical presentation ranges from salivation to depression, dyspnea, and asymptomatic to mild fever, as well as abortion and death^1^. According to the phylogenetic analysis of the more variable region of the BTV genome (Seg-2 region), at least 26 distinct serotypes have been identified around the World, each of which is able to cause disease^5^. In South America, BTV serotypes 1 to 4, 6 to 10, 14, 17 and 24 have been found previously in Argentina^6^, Brazil, Colombia, Guyana, and Peru^7^. In Ecuador the identified serotypes in cattle are 9, 13 and 18^8^.

Among wild species, collared peccaries (*Tayassu tajacu*) have been found to be infected in Brazil^9^ and Peru, marsh deer (*Blastocerus dichotomus*), pampas deer (*Ozotoceros bezoarticus*) and tapir (*Tapirus terrestris*) in Brazil^10^; grey brocket (*Mazama gouazoubira*) in Bolivia, guanaco (*Lama guanicoe*) and vicuna (*Vicugna vicugna*) in Argentina^11^. Currently, there is no available information on BT in wildlife in continental Ecuador and the virus is absent in the Galapagos islands^12^.

According to the WOAH, midges (genus *Culicoides*) are the only significant competent vector of BTV. They are also vectors of Vesiculo virus (vesicular stomatitis)^13^, Schmallenberg virus, African horse sickness virus, Aino virus and Akabane virus^14^. As a vector-borne disease, the natural distribution and prevalence of BT are governed mainly by ecological factors modulating vector populations (e.g., rainfall, temperature, humidity, and soil characteristics).

Transmission mainly occurs through the bite of infected midges (females)^15^. BTV requires a minimum temperature between 10 and 15 °C to replicate inside the *Culicoides* vector, as RNA polymerase activity is positively influenced by temperature^2^. Higher temperatures may increase the biting rate, favouring vector host transmission^15^. The temperature that maximises the chance of a midge surviving and consuming a blood meal is 23 °C, while 13 °C is the temperature that results in the greatest expected number of lifetime midge bites^16^. The inventory of *Culicoides* fauna in Ecuador comprises 70 species, including *C. insignis and C. paraensis*^17^.

Rainfall is also a determinant of the survival and activity of midges, and the abundance of the vector is often related to rainfall (and suitable temperatures). After rainfall, their feeding frequency changes^18–20^. Rainfall also governs the availability of larval habitat, survival, and dispersal of adults. The pupae of most species will float if submerged; however, the pupae of some species, such as *C. imicola*, can be drowned if inundated^20^.

The long-distance spread can also be attributed to animal movements in the case of insufficient veterinary control. Little evidence for contact transmission has been found only in goats, sheep and deer, and transplacental infection has been reported in cattle, sheep, and elk^14^.

Knowledge of BTV dispersion in Latin America is mostly limited to serological surveillance and a few molecular characterization reports. Additionally, risk analysis and systematic surveillance, which could guide prevention and control strategies are lacking. In Ecuador, BTV is not targeted by a specific surveillance approach but is included as a differential diagnosis of vesicular diseases in the foot and mouth disease (FMD) surveillance programme. Serological findings have been reported through the national surveillance system, scientific literature^8,12,21^, and international reporting systems since 2015 (https://wahis.woah.org/#/home/).

In this paper, we present the first analysis of ecological factors, such as temperature and rainfall dependency, in relation to past detections of antibodies against BTV in Ecuador. We hereby provide scientific grounds for future targeted surveillance and control strategies addressing BT.

## Results

The results reveal widespread evidence of BTV exposure, geographically matching favourable vector ecosystems within a temperature range of 12–32 °C. We explored the dependency of bioclimatic variables on natural regions and the distribution of the serological evidence across provinces. The variables most relevant for predicting high-risk areas for BT include cattle population, maximum temperature of the warmest month, minimum temperature of the coldest month, temperature seasonality, and precipitation of the driest month.

### Descriptive analysis of surveillance for BTV in Ecuador

**Fig. 1:**
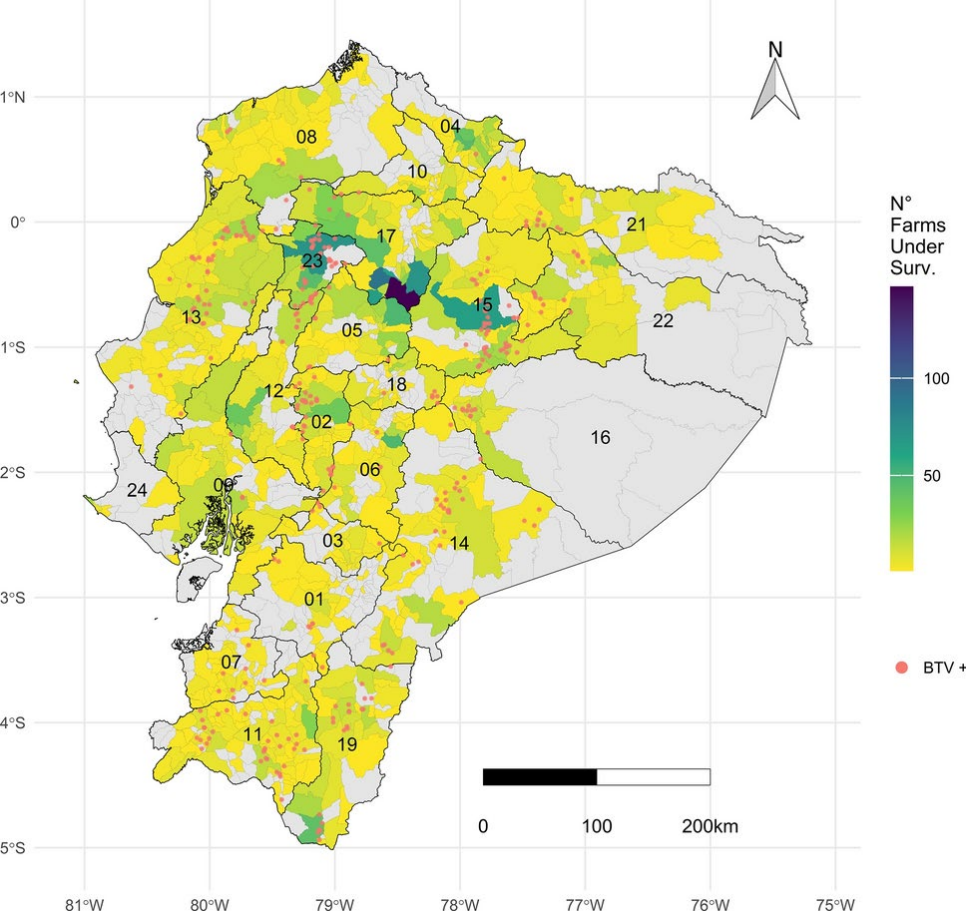
Surveillance of bluetongue in Ecuador from 2014 to 2022. BTV seropositive farms are represented by red dots. The map is divided by administrative levels: Provinces with bold black boundaries and IDs, and Parishes with grey boundaries. The IDs match the names of the provinces in Table 2. Parishes are filled with a gradient colour indicating the number of farms under surveillance that reported disease suspicion to the NVS (Galapagos islands are omitted).

Surveillance of BTV has been carried out by the National Veterinary Service (NVS) within the active and passive national surveillance strategy since the first official occurrence in the country in 2014 (https://wahis.woah.org/, accessed on 01 June 2023). General (passive) surveillance is based on the reporting of clinical symptoms such as fever, vesicular lesions, a drop in performance or abortions. Between November 2014 and December 2022, a total of 5,015 suspicious reports were received from 647 parishes, accounting for 62.2% of Ecuador’s 1,040 parishes and 100% of its 24 provinces. The map in Fig. 1 illustrates the political divisions of Ecuador, highlighting both the first-level administrative divisions (provinces), and the third-level administrative divisions (parishes), where the analysis was conducted in greater detail.

The suspicious reports were examined by official veterinarians considering the clinical manifestations and farms records. Differential diagnoses included brucellosis, infectious bovine rhinotracheitis, anaplasmosis, bovine viral diarrhea, leucosis, BT, neosporosis, and FMD.

Following notification, out of the 5,015 reports, 381 farms with BT compatible signs were sampled for laboratory confirmation. Of these, 324 farms were seropositive for BTV. The monthly temporal distribution of analyzed samples and farms is presented in Supplementary Figure S1 online. Most of the affected farms were cattle farms (n = 378; 99,2%), followed by sheep (n = 2; 0,5%) and goat farms (n = 1; 0,3%). From a total of 5,901 samples obtained from these farms, a total of 5,161 animals had positive diagnoses for BTV antibodies (Table 1). BTV antibodies were not detected in other species, such as Andean camelids (llamas), or buffalo. Wildlife species were not tested in the analysed period.

**Table 1:** Reports, positive diagnoses, and apparent prevalence of bluetongue virus antibodies in cattle, sheep, and goats in Ecuador between 2014 and 2022.

Year	Positive farms/Sampled farms				Positive animals/Sampled animals				Apparent prevalence (%)			
	C	S	G	**T**	C	S	G	**T**	C	S	G	**T**
2014	1			**1**	43			**43**	100			**100**
2015	34/36	0/1	1/1	**35/38**	1,001/1,024	0/48	5/15	**1,006/1,087**	97.8		33.3	**92.5**
2016	72/81	1/1		**73/82**	1,577/1,767	10/15		**1,587/1,782**	89.2	66.7		**89.1**
2017	48/55			**48/55**	773/850			**773/850**	90.9			**90.9**
2018	123/149			**123/149**	1,345/1,590			**1,345/1,590**	84.6			**84.6**
2019	23/28			**23/28**	176/253			**176/253**	69.6			**69.6**
2020	15/19			**15/19**	157/187			**157/187**	84.0			**84.0**
2021	6/8			**6/8**	74/109			**74/109**	67.9			**67.9**
2022	0/1			**1**	0/17			**0/17**	0			**0.0**
Total	322/378	1/2	1/1	**324/380**	5,146/5,823	10/63	5/15	**5,161/5,901**	88.4	15.9	33.3	**87.5**

Species: C = Cattle; S = Sheep; G = Goats.

Total values are in bold (T).

The occurrence of BTV antibodies on farms increased from 2016 (n = 82; 21.6%) to 2018 (n = 149; 39.2%), peaked in 2016, and significantly declined in 2019–2022 (Table 1).

Differences in the number of farms testing positive for BT antibodies and the apparent prevalence rates were evident among provinces. Within the coastal region, Guayas, Esmeraldas and Los Rios, exhibited particularly elevated rates, similar to Morona, Orellana and Zamora in the Amazon region. Conversely, provinces in the highland region such as Carchi, Chimborazo and Azuay exhibited the lowest rates. Specifically, Napo, Loja and Santo Domingo provinces recorded the highest number of farms with positive test results (Table 2, Fig. 1).

**Table 2:** Overview of farms with positive diagnoses and the apparent on-farm prevalence of BTV antibodies in ruminants between 2014 and 2022 in different provinces of Ecuador.

Province	ID	Positive farms	Animals on the farms	Samples	BTV +	Apparent prevalence
Morona Santiago	14	29	705	657	657	100.0
Orellana	22	19	444	293	293	100.0
Guayas	09	3	207	35	35	100.0
Zamora Chinchipe	19	21	519	413	410	99.3
Esmeraldas	08	7	483	124	123	99.2
Los Rios	12	9	1972	357	354	99.2
Sucumbios	21	12	408	257	253	98.4
El Oro	07	9	286	115	111	96.5
Santo Domingo	23	40	6,719	836	805	96.3
Napo	15	48	746	526	487	92.6
Pastaza	16	13	256	230	205	89.1
Cotopaxi	05	10	83	83	69	83.1
Bolivar	02	37	568	426	345	81.0
Loja	11	40	803	477	364	76.3
Manabi	13	14	3,267	199	151	75.9
Pichincha	17	13	718	348	253	72.7
Imbabura	10	16	574	95	60	63.2
Canar	03	9	531	133	76	57.1
Tungurahua	18	9	118	106	55	51.9
Azuay	01	10	142	140	49	35.0
Chimborazo	06	8	66	27	5	18.5
Carchi	04	5	41	41	1	2.4
Total		381	19,656	5,918	5,161	76.7

### Ideal temperature conditions for vector survival

**Fig. 2:**
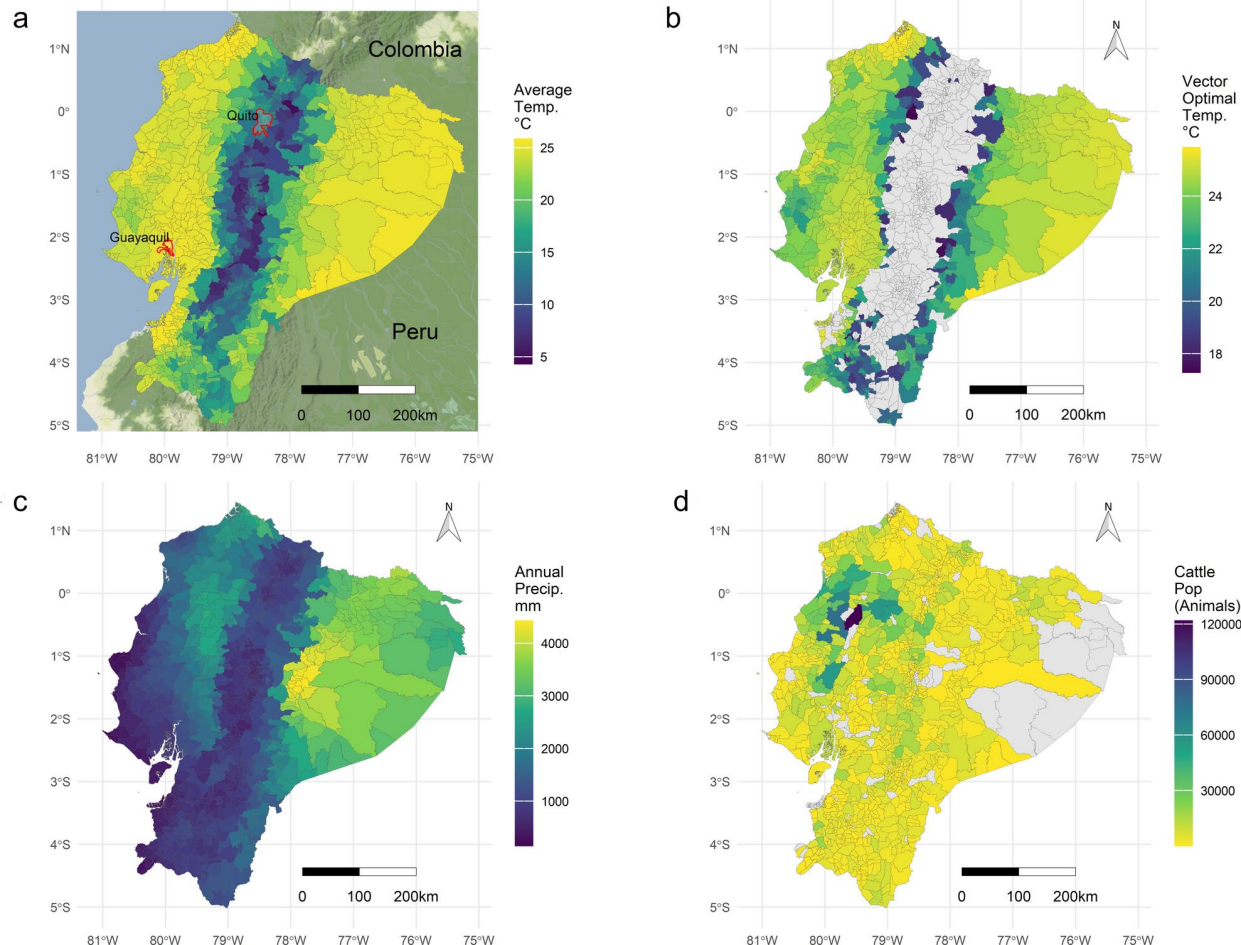
Overview of environmental characteristics and host species related to the bluetongue presence in Ecuador at the parish level from 2014 to 2022. (**a**) Annual mean temperature. Metropolitan areas of the largest cities (highlighted in red): Guayaquil is in the Coastal region, and Quito (the capital) is situated in the Highland region. (**b**) Culicoides ideal survival temperature. The optimal temperature range for vectors corresponds to temperatures a minimum of 12 °C and a maximum of 26 °C. Parishes with the least likely presence of Culicoides outside this optimal temperature range are shown in grey. (**c**) Annual mean precipitation in mm shows, highest values in the central Amazon region and the north-western Coastal region. (**d**) Cattle population in 2023 shows the highest populations observed in parishes corresponding to Manabi and Santo Domingo provinces.

Although Ecuador is located in a tropical region crossed by the equator, extensive areas experience cold temperatures. This is mainly due to the Andean Mountains, which extend in a north–south direction and reach altitudes exceeding 6000 m. Major cities, such as the capital Quito, are situated at altitudes above 2500 m (Fig. 2a). Livestock populations, particularly dairy farms, are concentrated in the Andean highlands, where annual mean temperatures remain below 15 °C. These areas can be identified in Fig. 2d as having higher parish cattle population along the north–south axis of the highlands. Additionally, even higher cattle population are observed in the northwestern coastal parishes, where beef production is predominant.

The mean temperature of parishes where BTV antibodies were detected was 20.62 °C (IQR = 5.2 °C). In contrast, the mean temperature where BTV antibodies were not detected was 18.37 °C (IQR = 11.4 °C). It is possible to observe higher temperatures in the Eastern (Amazon) region and Western (coastal) region under ideal conditions for the survival and proliferation of *Culicoides spp.* vectors.

The analysis of parishes with optimal survival conditions for *Culicoides spp.* revealed that 54% (561 of 1040) of the parishes had ideal temperature conditions. These parishes are highlighted in Fig. 2b, while the remaining parishes, shaded in grey, indicating lower temperature areas. In total, 73% (180.800 km^2^) of the national territory, provided optimal survival conditions, defined as minimum temperature of 12 °C and maximum of 32 °C. When increasing the minimum temperature by 2.6 °C, as the expected warming over humid lands^22^ due to global warming, there could be an increase up to 80% (198.188 km^2^) of the national area and 61% (637 of 1040) of the parishes providing optimal survival conditions. The location of the posible 76 new parishes can be found as Supplementary Fig. S4 online.

The mean annual precipitation of parishes where BTV antibodies were detected was 1983 mm (IQR = 1576 mm), whereas in parishes without BTV antibody detection, it averaged 1373 mm (IQR = 853.79 mm). Higher precipitation levels are observed in the Amazon region and in the central-northwestern coastal region, which may contribute to the survival and proliferation of the *Culicoides spp.* vectors (Fig. 2c).

The minimum total parish precipitation with registered outbreaks was 513 mm. In Spain, annual rainfall levels favourable for the survival of midges were approximately 600 mm, while in Africa, they ranged between 300 and 750 mm^23^. Parishes with lower annual rainfall are located along the Pacific coast, where no outbreaks were recorded despite falling within the suitable temperature range, likely due to insufficient precipitation.

The cattle population in Ecuador was analysed, considering that BTV occurrence is strongly dependent on host abundance. According to the 2023 vaccination campaign against FMD, 4.6 million cattle were vaccinated. The province of Manabi (northwestern coastal region) has the largest cattle population, with 0.97 million animals. Pichincha (north-central highlands, including the capital Quito) and Esmeraldas (northwestern coastal province bordering Colombia) follow with 0.37 million and 0.36 million cattle, respectively (Fig. 2d).

The median cattle population per parish was 2,323.6 (Q1: 838.8, Q3: 5,353.8), with a maximum of 121,853.0 in Manabi, which host the second Ecuador’s largest livestock market, *El Carmen*. The median cattle density per parish (animals/km^2^) was 22.26 (Q1:8.32, Q3:47,28), with the highest density recorded in Tungurahua (central highlands, *Pelileo*) at 808.75 animals/km^2^. A cattle density map at the parish level is provided in Supplementary Figure S3 online.

The provinces with the highest cattle densities were Carchi (bordering Colombia), Tungurahua, and Cotopaxi in the central highlands, with densities of 81.2, 72.1 and 63.6 animals/km^2^, respectively (Table 3).

**Table 3:** Cattle population density and annual minimum temperature by province in Ecuador in 2023.

Province	Cattle population	Density (animals/Km2)	Min Temp. (C')
Manabi	977,503	42.37	18.63
Pichincha	375,447	41.52	7.3
Esmeraldas	368,251	33.77	20.73
Guayas	297,254	26.07	19.13
Chimborazo	260,321	46.75	5.07
Cotopaxi	258,187	63.64	6.5
Santo Domingo	209,653	63.08	17.65
Loja	195,233	20.42	12.85
Azuay	176,345	30.11	7.59
Morona Santiago	165,256	16.6	14.64
Carchi	159,168	81.23	7.4
Bolivar	157,692	40.07	9.18
El Oro	156,984	35.81	17.03
Canar	142,555	63.2	7.77
Zamora Chinchipe	135,054	17.13	13.75
Tungurahua	132,964	72.07	5.81
Sucumbios	129,918	13.5	17.24
Imbabura	104,340	31	7.45
Los Rios	83,768	11.47	19.32
Orellana	75,939	18.44	19.13
Napo	49,519	8.28	10.34
Pastaza	25,345	9.94	16.85
Santa Elena	19,693	4.46	19.21
Total	4′656,389	34.38	

### Modelling bioclimate variables

Each pair of bioclimatic variables included in the model had a correlation of < 0.54. Of the 13 variables analysed, six were significant in distinguishing parishes where outbreaks were present. The model demonstrated a low error rate (0.14), a high degree of adjustment (Hosmer–Lemeshow test = 0.9) and an acceptable fit (AUC = 0.77) (Table 4).

**Table 4:** Logistic model results.

Variable	β	OR	CI95		Pr( >|Z|)	Sig
Precipitation of wettest month	6.56E-03	1.007	1.004	− 1.009	5.69E-08	***
Population of cattle	6.52E-05	1.000	1.000	− 1.000	1.55E-06	***
Max temp of warmest month	3.08E-01	1.360	1.156	− 1.601	2.09E-04	***
Min temp of coldest month	− 2.76E-01	0.759	0.648	− 0.889	6.17E-04	***
Temperature seasonality	− 1.55E-03	0.998	0.997	− 0.999	1.94E-03	**
Precipitation of driest month	2.38E-03	1.002	1.000	− 1.005	9.10E-02	

AIC: 744, R2:0.217, AUC: 0.78, error: 0.14, Hosmer–Lemeshow: 0.9

The maximum temperature of the warmest month increased the odds of vector presence in Ecuadorian parishes (OR = 1.36); whereas the minimum temperature of the coldest month had the opposite effect (OR = 0.76) acting as a protective factor within the ideal temperature range (Fig. 2b). This highlights the contrast between the colder Andean zones and the warmer coastal and Amazonian regions. The precipitation of the wettest month, cattle population, and other variables were highly significant in the model; however, their individual effects were small (low odds ratios).

Collinearity analysis revealed values below 1.8 for all analysed variables. One observation had a significant influence on model fitting, as identified by the Bonferroni outlier test (*p* = 0.004). Additionally, no correlation was identified between the residuals.

**Fig. 3:**
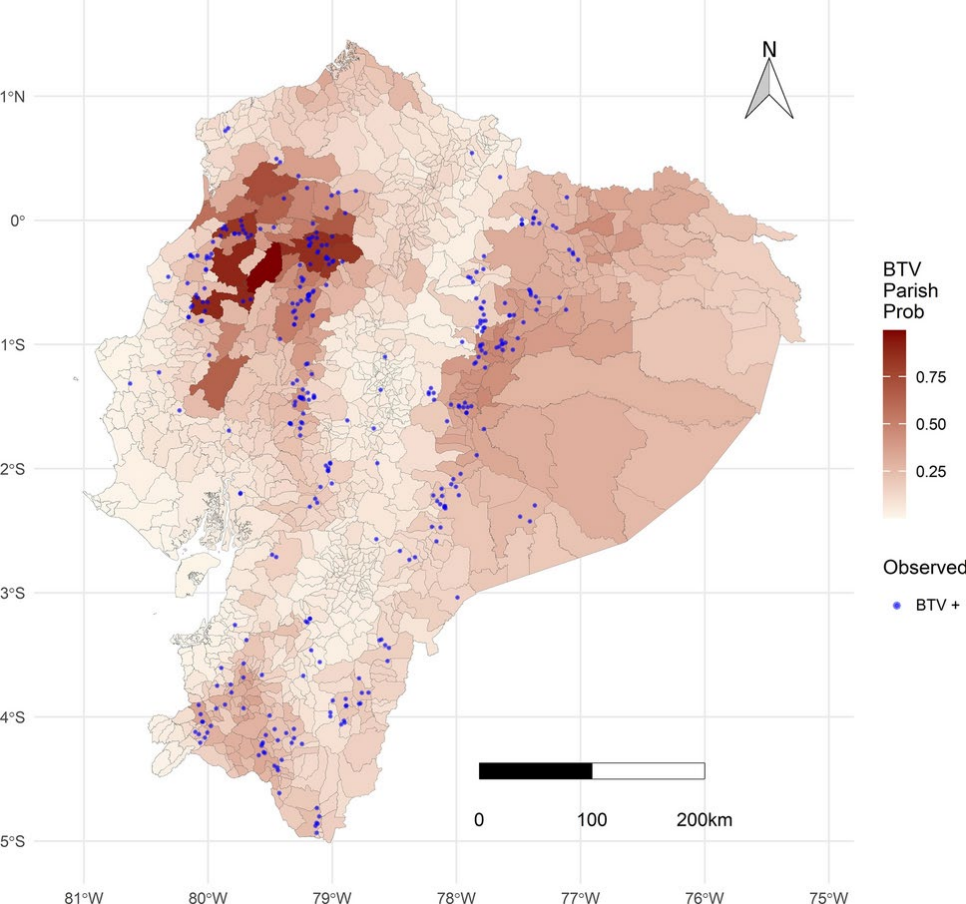
Probability map of BTV occurrence in Ecuador. The model, based on bioclimatic variables and host population, revealed higher probabilities in areas with elevated temperatures. Low precipitation, particularly along the central Pacific coast, acts as a protective factor, contrasting with the more humid areas of the northern Pacific coast. Lower probabilities are observed in the highlands. Farms with serological findings of BTV are indicated by blue dots.

The suitability of sites in Ecuador for BTV occurrence, based on bioclimatic variable modelling, is shown in Fig. 3. The analysis identified a high probability of BTV occurrence in transition zones from the highlands to the Amazon, particularly in the central-eastern provinces of Napo and Orellana, as well in the central-northwestern (Santo Domingo) and southern (Loja) provinces. Further considerations regarding data and modelling limitations are available in Supplementary Note S5 online.

## Discussion

Widespread BTV exposure in Ecuadorian cattle emphasises the need for enhanced surveillance systems, given the likely underestimated impact of bluetongue disease (BT) by both producers and the veterinary service. Climatic conditions significantly influence vector abundance, highlighting the necessity for targeted control measures. This analysis, the first of its kind for an Andean country with diverse ecosystems, can inform initial strategic approaches for risk-based surveillance and control measures, adopting a comprehensive One Health framework.

Given the limited available data on BT prevalence in Latin America, the overall impact of the disease is most likely underestimated by producers and veterinary services alike. Considering the effects of BTV on cattle production—such as a relative reduction in milk production, postponed gestation, no gestation, and abortion^24^—there is a clear need for scientific evidence to support a better understanding and control BT in the region.

The evidence of widespread BTV exposure in Ecuadorian cattle was derived from untargeted passive surveillance within brucellosis and FMD monitoring programmes. A key limitation is the absence of a BT case definition in the NVS system, as BT is currently addressed only as a differential diagnosis within the general surveillance. This omission likely leads to significant underestimation of the true disease burden.

The presented analysis of prevailing climatic conditions and their expected impact on vector abundance is in line with current knowledge^25^. The mean parish temperature at which the outbreaks occurred in this study was 20.1 °C, which is in line with other temperature-dependent transmission studies^26^ in which the temperature ranged from 15 to 26 °C. The identification and prioritization of parish, as performed in this and other paper^27^, could be valuable for informing animal health decision-making, identifying possible at-risk areas of spread, focusing on specific surveillance of BTV or even on animal movement to avoid the transport of positive animals^28^ and implementing preventive and control measures in large livestock markets^29^ around the country.

Temperature and precipitation are related to the biting rate of *Culicoides* spp., the time required for oogenesis, oviposition, the time needed to digest the blood meal, and the BTV replication rate^30^. Environmental conditions are one of the key aspects for implementing a successfully surveillance and control strategy. In this paper, we used average temperatures and rainfall, but further studies could improve the temperature resolution using monthly data to establish temporal and seasonal implications.

Several species of *Culicoides* described in Ecuador have public health implications because they have been reported as possible agents of skin zoonosis (*C. insignis, C. pachymerus, and C. paraensis*), filariasis (*C. pifanoi*), allergic dermatitis (*C. acotylus, C. fluvialis, and C. leopoldoi*) and mansonellosis (*C. guttatus*); others have been reported to carry DNA from *Leshmania brasiliensis* and *Amazonensis* (*C. foxi*, *C. insignis*, and *C. filarifer*)^17^. Giving rise to the importance of improving the knowledge of the vector not only for veterinarian interest but also as a public health concern and wildlife affectation that could be better addressed considering One Health approach.

Knowledge of the risk areas for BTV vectors could inform recommendations for vector control as an attempt to reduce virus transmission by reducing vector-host contact^31^, and implementing the use of sticky resting boxes as a tool for monitoring the presence of *Culicoides*^32^, insecticides^33,34^, are often discussed as options; however, their widespread implementation and effects must be carefully assessed for economic and environmental sustainability.

When considering the host factor, cattle population serves as a risk factor for BTV presence, but only as a cofactor with temperature-precipitation seasonality and annual precipitation levels that fall within the optimal range for *Culicoides* development. This is evident in parishes with high cattle population and densities but no seropositivity findings, particularly in the highland central zone where low temperatures prevail, showing that the climate factors are determinant. However, vaccination^35^ of susceptible cattle and small ruminants against circulating BTV serotypes presents a viable tool for protect livestock and avoid further spread^36,37^. The findings of this study can inform targeted vaccination strategies to mitigate the spread of BT, particularly in relation to the transport of animals from high-risk areas.

Currently, understanding the precise areas where *Culicoides* are most likely to be present, in relation to climate conditions such as temperature and precipitation, is of paramount importance. Furthermore, it is necessary to undertake further studies of elucidate the impact of climate change^38–40^, as rising temperatures will influence the distribution of vector habitats^41^. This will not only modify the current situation but also inform future predictions (see Supplementary Fig. S4 online).

In the South American context, it is crucial to explore the previously uncharted role of wildlife in virus maintenance^9,10^, including its effects on various populations^42^.

## Materials and methods

Passive surveillance data from the Veterinary Service were analysed from farms screened for BTV antibodies using c-ELISA. Descriptive analysis was then conducted on historical surveillance data. Ideal temperature conditions for Culicoides vector survival were assessed using bioclimatic variables; Finally, logistic regression determined the influence of environmental factors on parish BTV status.

### Surveillance data

We analysed the passive surveillance information of suspicious and confirmed reports from the Veterinary Service database from 2014 to 2022. The analysed dataset was obtained from the official system (www.sistemas.agrocalidad.gob.ec/sizse) accessed on 01.04.2023). The information was registered by official veterinarians accessing the institutional system when visiting the farms. Cadastral records are updated each semester when bovines are vaccinated against FMD, and movement records between farms and traders update the individual farm records. The farms were sampled by the official veterinarians according to internal procedures. Serum samples were screened for the presence of BTV antibodies using a bluetongue antibody test kit, c-ELISA (VMRD, Pullman WA, USA), which was performed according to the manufacturer’s instructions. The test was positive if the sample produced more than 60% inhibition. All samples were analysed in the national reference laboratory.

### Descriptive analysis of BTV surveillance in Ecuador

During the 2014–2022 period, the NVS collected information on vesicular and reproductive diseases and their differentials via passive surveillance. BTV was included in the differential diagnoses registering large datasets of information about the disease. We performed a descriptive epidemiological analysis on the available historical information.

### Ideal temperature conditions for vector survival

We analysed the relationships between the ideal survival temperature ranges of *Culicoides spp.* and the maximum and minimum parish temperatures at the best geographic resolution (parish), to identify the geographic locations that provided optimal survival conditions for the *vector.*

The ideal temperature ranges are defined by a minimum temperature threshold of 12 °C, which maximizes the likelihood of a BTV-infected midge surviving its extrinsic incubation period^16^, as well as the apparent absence of virus replication below 15 °C^20^. Maximum temperatures were set at 34 °C, representing the anticipated limit of infective life^43^ and 33 °C, beyond which negative effects on oviposition^44^.

Bioclimatic variables were obtained at a spatial resolution of 2.5 arc-minutes (~ 5 km^2^); and the extracted values from a raster object at the locations of the spatial vector data were analysed using Raster (R package V3.5)^45^. The official map layers were obtained from the Institute of Statistics and Census of Ecuador (http://www.geoportaligm.gob.ec/, accessed on 1 February 2023). The temperature and precipitation data were extracted from WorldClim (https://worldclim.com/, accessed on 1 January 2023). Analyses were computed using R V.4.3.2 (https://cran.r-project.org/, accessed on 1 February 2025).

### Modelling bioclimate variables

To determine the association of environmental variables on the *Culicoides* parish distribution, we considered 12 bioclimatic variables (see Supplementary Fig. S2 online) as risk factors in the model and the host population (cattle). Climate data were obtained from the spatial resolution climate surfaces for global land areas^46^. The variables included in the model (Table 5) were chosen based on their correlation values |r|< 0.7. Variance inflation factors were calculated into the model to avoid multi-collinearity of environmental variables |vif|< 10^47^. The evaluation of variables was based on the association of each explanatory variable with the binary outcome parish BTV status (antibodies detected yes/no) using logistic regression^48^. We used a manual backwards exploratory selection of variables and then a forward stepwise selection^49^. The goodness of fit of the final model was measured using conditional R2, ROC and Hosmer–Lemeshow tests^50^.

**Table 5:** Variables analysed in the model.

Variables	Description
bio_1	Annual mean temperature
Bio_4	Temperature seasonality (sd × 100)
Bio_5	Max temperature of warmest month
Bio_6	Min temperature of coldest month
Bio_8	Mean temperature of wettest quarter
Bio_9	Mean temperature of driest quarter
Bio_12	Annual precipitation
Bio_13	Precipitation of wettest month
Bio_14	Precipitation of driest month
Bio_15	Precipitation seasonality (sd × 100)
Bio_18	Precipitation of warmest quarter
Bio_19	Precipitation of coldest quarter

## Supplementary Information

Supplementary Information.

## Data Availability

The datasets generated during and/or analysed during the current study are available in the BTV [BTV-EC] repository. Aggregated information is presented as raw data cannot be publicly disclosed due to legal restrictions protecting the private information of Ecuadorian producers.
