# Supplementary material for: Linking vector favourable environmental conditions with serological evidence of widespread bluetongue virus exposure in livestock in Ecuador
Source: Sci Rep. 2025 Apr 24;15:14382. doi: 10.1038/s41598-025-95918-7 (PMC12022061; doi:10.1038/s41598-025-95918-7)
Supplement: Supplementary file 1 — Supplementary Information. [file 41598_2025_95918_MOESM1_ESM.pdf]

Linking vector favourable environmental conditions with serological evidence of widespread Bluetongue virus exposure in livestock in Ecuador

Alfredo Acosta*[1,2], Maritza Barrera[4], David Jarrín[3], Alexander Maldonado[3], Johanna Salas[3], Guilherme Camargo[2], Beatriz Mello[2], Alexandra Burbano[3], Euclides DelaTorre[3], Bernd Hoffman[1] and Klaas Dietze[1]

[1] Friedrich-Loeffler-Institut. Greifswald. Germany.

[2] Preventive Veterinary Medicine Department, School of Veterinary Medicine, University of São Paulo, São Paulo, Brazil.

[3] Agencia de Regulación y Control Fito y Zoosanitario-Agrocalidad. Quito. Ecuador.

[4] Veterinary Department, Faculty of Veterinary Sciences, Universidad Técnica de Manabí, Portoviejo, Ecuador.

Address for correspondence: Alfredo Acosta. Department of Epidemiology, Disease Surveillance and Risk Assessment. Swedish Veterinary Agency, SVA. Address: Ulls väg 2B. 75189. Uppsala, Sweden. e-mail: alfredo.acosta@sva.se

KEYWORDS: bluetongue; surveillance system; culicoides; risk analysis; policy.

Supplementary Information

Considering the number of samples collected, the detection of BTV antibodies was more intense from May 2015 to July 2020, with positive antibody diagnostics exceeding 87%. The average number of monthly reports from 2015 to 2021 was 5.23, with peaks above 15 in January 2016 and July 2018. Additionally, 64.58% of the weeks confirmed cases (Fig. S1).

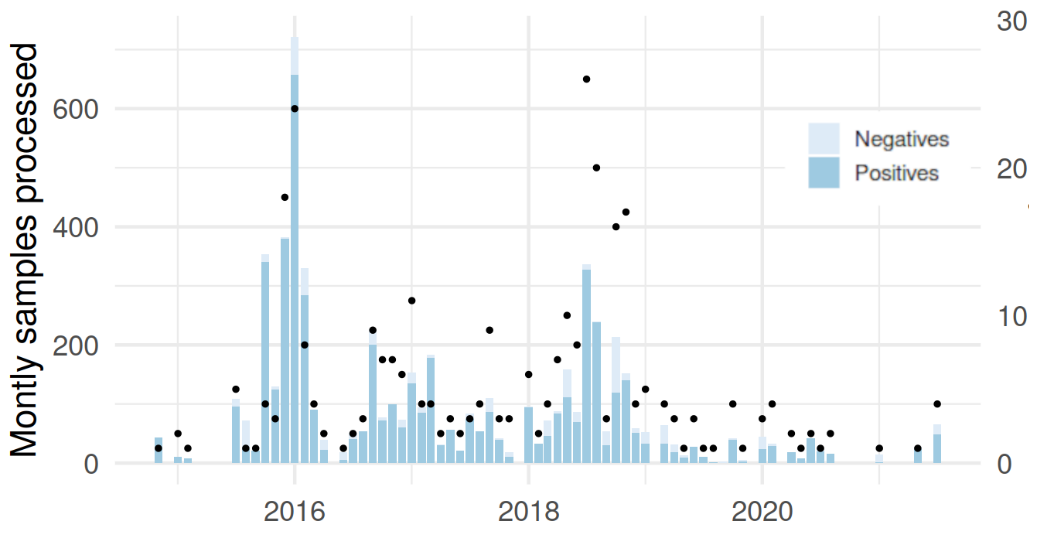

Supplementary Figure S1. Temporal distribution of analysed samples (bars) and farms (dots) during the period 2014-2021 in Ecuador. Second axis scale corresponds to the number of farms (dots) being analysed.

The Bioclimatic variables analysed in the model are presented on Supplementary Fig.S2. The names are in concordance to Table 5 of the article. It is possible to observe the influence of the Andes Mountains (strip running from north to south) generating different

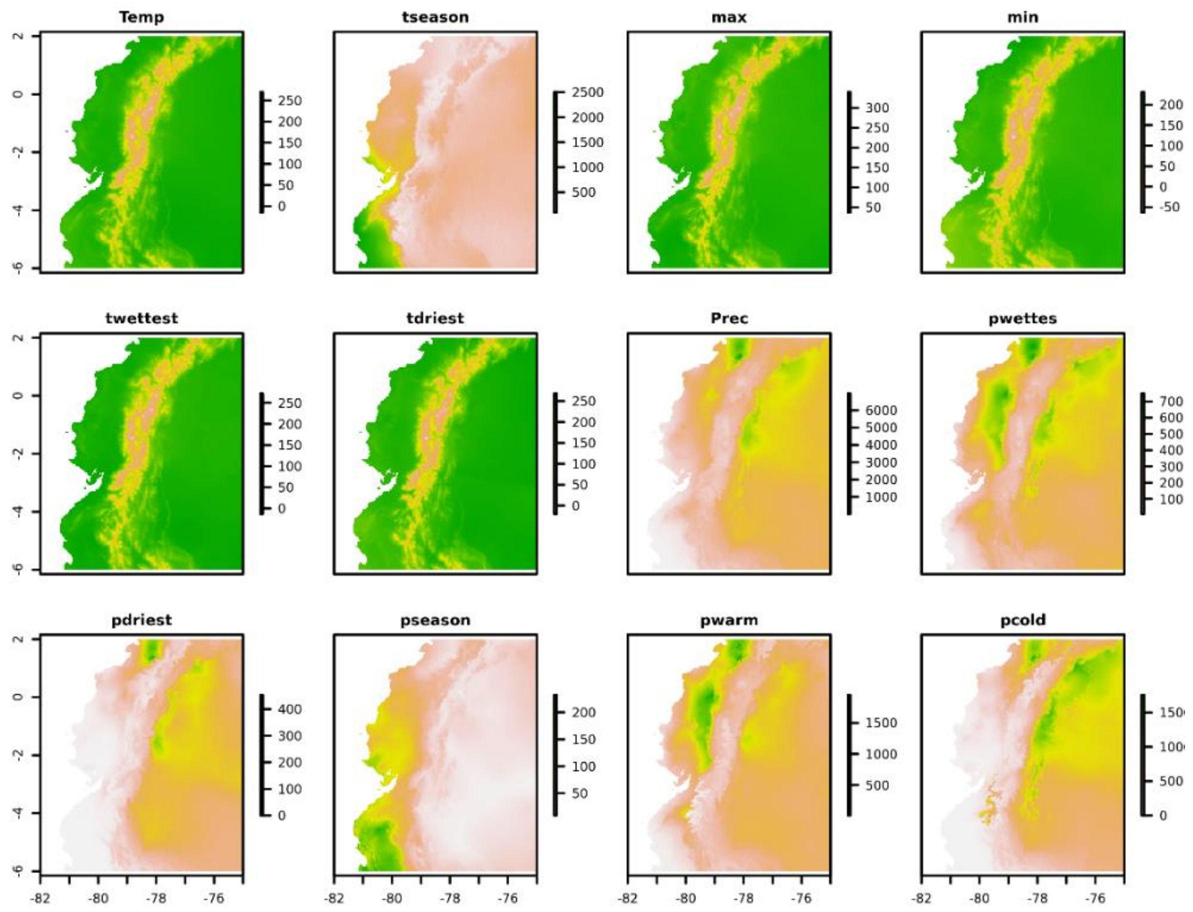

Supplementary Figure S2. Visualization of bioclimatic variables used in the model of BTV in Ecuador.

The parishes exhibiting higher cattle densities are not confined solely to the coastal regions, as illustrated in the total population maps. These high-density areas are also found in the Highlands, where the primary focus is on milk production. Conversely, in the coastal regions, the emphasis shifts towards meat production (Sup. Fig. S3 and S2).

A temperature rise of 2.7 degrees could shift the geographical range of ideal conditions for vectors in 75 new parishes and potentially facilitating their introduction into new areas. Parishes situated in the Southern Highland region, such as the province of Loja and Zamora, especially those at lower altitudes, might experience notable impacts. This effect could extend across transition zones from the Andean Mountains to the coastal and Amazon regions (Sup. Fig. S3).

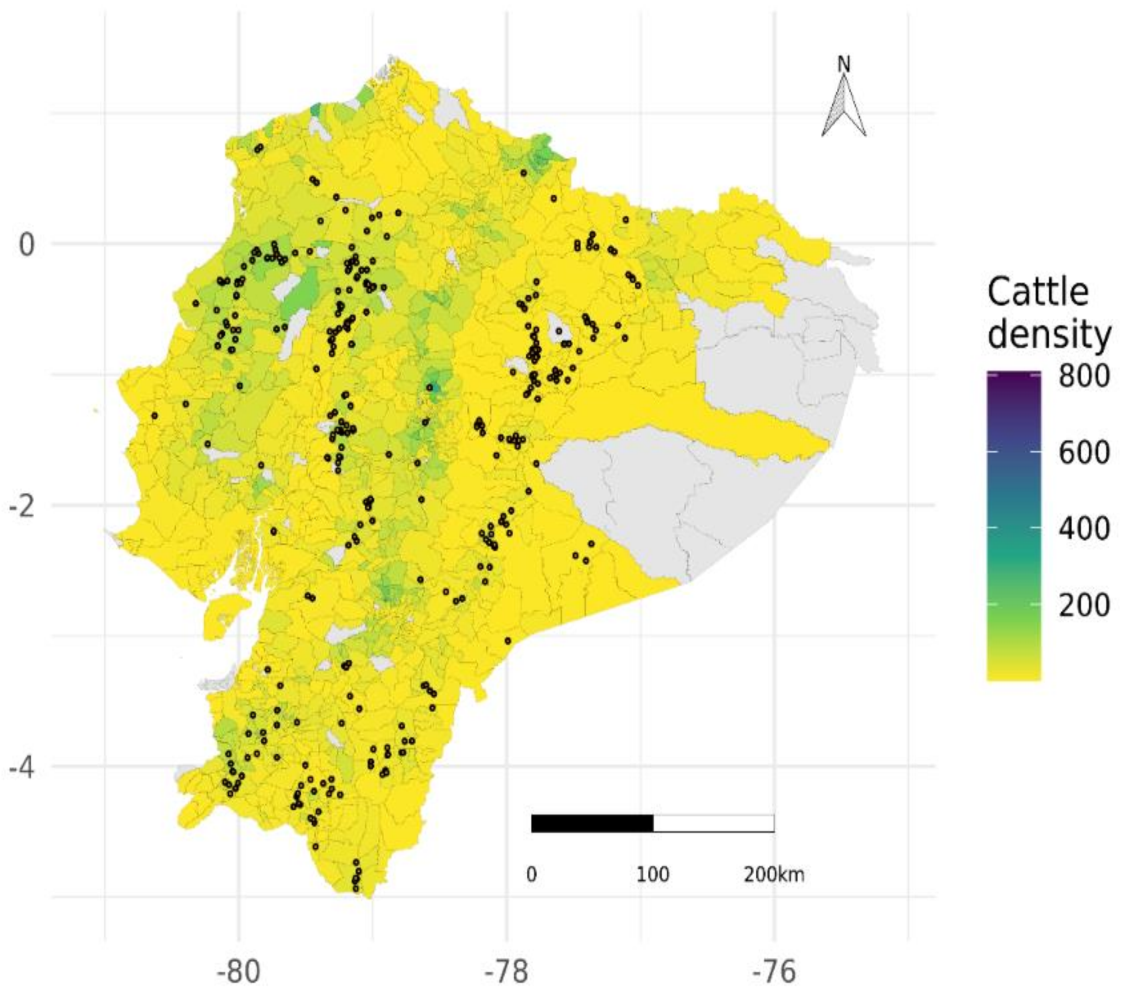

Supplementary Figure S3. Cattle density map, parishes are coloured according to the number of animals vaccinated against FMD in 2023, points showing the location of BTV + serological findings between 2014 and 2022.

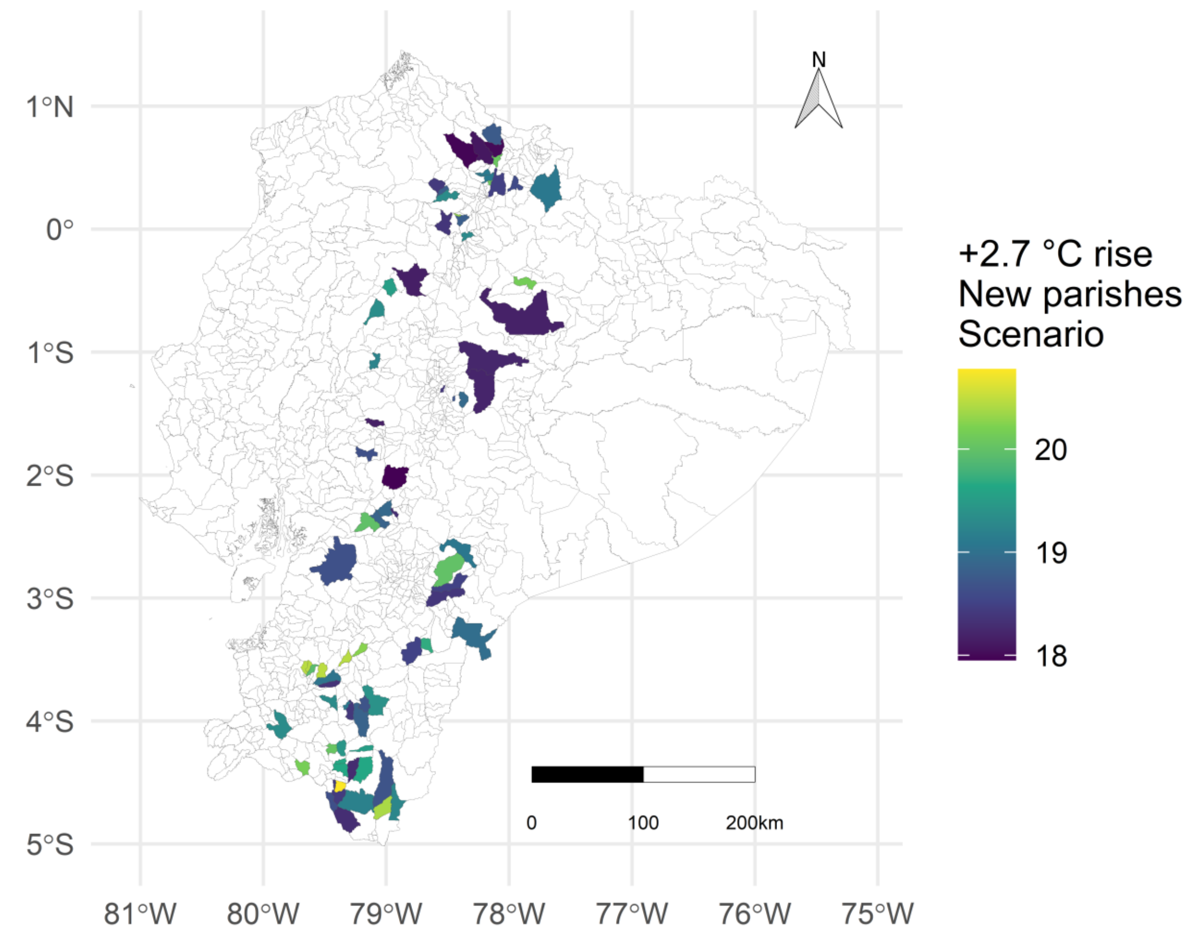

Supplementary Figure S4. Impact of 2.7 degrees increase in optimal temperature range for Culicoides due to global warming scenario. On the map it is possible to locate 76 new parishes with ideal conditions for vectors in Ecuador.

Supplementary note S5. Data limitations and further methodology remarks.
The decision to use parish-level data rather herd-level data was primarily influenced by limitations in the available dataset at the time of analysis. While herd-level data could potentially enhance the statistical power of the model, it also introduces greater complexity and uncertainty due to variability in data quality and availability. Given that the datasets originated from historical veterinary surveillance efforts, inconsistencies in case definitions and data collection methods over time posed additional challenges.

Aggregating data at the parish level offered several advantages. It simplified the model, reducing the risk of overfitting that could arise from missing or inconsistent herd-level data. Furthermore, parish-level aggregation helped smooth of anomalies that might be present at herd level. Importantly, this approach also aligned with the practical needs of the National Veterinary Service by facilitating the development of a risk map at the smallest administrative scale already used by the NVS, thereby supporting targeted surveillance and control efforts. Nonetheless, parish level limits the resolution of our findings.

Future research could benefit from recent improvements in herd-level data collection in Ecuador, including the registration and verification of geographic coordinates and map projections. These advancements will enable more refined spatial analyses and epidemiological models, further enhancing our understanding of Bluetongue virus risk and transmission patterns.
